# Altered T Lymphocyte Proliferation upon Lipopolysaccharide Challenge *Ex Vivo*

**DOI:** 10.1371/journal.pone.0144375

**Published:** 2015-12-07

**Authors:** Fanny Poujol, Guillaume Monneret, Alexandre Pachot, Julien Textoris, Fabienne Venet

**Affiliations:** 1 BioMérieux, Joint Research Unit, Hospices Civils de Lyon—bioMérieux, Hôpital Edouard Herriot, Lyon, France; 2 Immunology Laboratory, Hospices Civils de Lyon, Hôpital Edouard Herriot, Lyon, France; 3 Anesthesiology and Intensive care department, Hospices Civils de Lyon, Hôpital Edouard Herriot, Lyon, France; Universitatsklinikum Freiburg, GERMANY

## Abstract

**Context:**

Sepsis is characterized by the development of adaptive immune cell alterations, which intensity and duration are associated with increased risk of health-care associated infections and mortality. However, pathophysiological mechanisms leading to such lymphocyte dysfunctions are not completely understood, although both intrinsic lymphocyte alterations and antigen-presenting cells (APCs) dysfunctions are most likely involved.

**Study:**

The aim of the current study was to evaluate whether lipopolysaccharide (LPS, mimicking initial Gram negative bacterial challenge) could directly impact lymphocyte function after sepsis. Therefore, we explored *ex-vivo* the effect of LPS priming on human T lymphocyte proliferation induced by different stimuli.

**Results:**

We showed that LPS priming of PBMCs reduced T cell proliferative response and altered IFNγ secretion after stimulation with OKT3 but not with phytohaemagglutinin or anti-CD2/CD3/CD28-coated beads stimulations. Interestingly only LPS priming of monocytes led to decreased T cell proliferative response as opposed to LPS priming of lymphocytes. Importantly, LPS priming was associated with reduced expression of HLA-DR, CD86 and CD64 on monocytes but not with the modification of CD3, CTLA4, PD-1 and CD28 expressions on lymphocytes. Finally, IFNγ stimulation restored monocytes accessory functions and T cell proliferative response to OKT3.

**Conclusion:**

We conclude that LPS priming does not directly impact lymphocyte functions but reduces APC’s capacity to activate T cells. This recapitulates *ex vivo* indirect mechanisms participating in sepsis-induced lymphocyte alterations and suggests that monocyte-targeting immunoadjuvant therapies in sepsis may also help to improve adaptive immune dysfunctions. Direct mechanisms impacting lymphocytes being also at play during sepsis, the respective parts of direct versus indirect sepsis-induced lymphocyte alterations remain to be evaluated in clinic.

## Introduction

Septic syndromes represent a major healthcare problem worldwide accounting for a high number of deaths every year [1,2].

Sepsis is characterized by the development of a phase of immunosuppression affecting both innate and adaptive immunity. In particular, T cells are deeply altered. After a massive apoptosis, the remaining T cells are anergic, display lower proliferation and secretion of pro-inflammatory cytokine after *ex-vivo* stimulation. In addition, circulating lymphocytes in septic patients present an exhausted phenotype, characterized by lower levels of CD3 and co-stimulatory molecule, increased expression of co-inhibitory receptors such as PD-1 (programmed cell death receptor-1) or CTLA-4 (cytotoxic T lymphocyte associated protein 4) [3,4]. Many studies have demonstrated an association between intensity and length of sepsis-induced T cell anergy and/or lymphopenia and increased risk of HAI (healthcare associated infections) and mortality [5,6]. This constitutes the rational for innovative therapeutic interventions (such as rhIL-7 (recombinant human interleukin-7) or anti-PD-1/PD-L1 (PD-1 ligand) antibodies) targeting these lymphocyte alterations that are now considered in the treatment of septic patients [7].

However, pathophysiological mechanisms leading to such lymphocyte dysfunctions are not completely understood. In particular, a potential direct effect of the initial infectious challenge on lymphocyte effector functions has never been evaluated in the context of sepsis.

Therefore, the aims of the current study were to explore lymphocyte functions after LPS (lipopolysaccharide, mimicking initial Gram negative infection) challenge *ex-vivo* in order to improve our understanding of sepsis-induced T cell alterations pathophysiology and to establish a lymphocyte functional tests that would help patients’ stratification in clinical trials. Therefore, we evaluated the effect of LPS priming on lymphocyte proliferation and cytokine production induced by different stimuli *ex-vivo*.

## Material and Methods

### Healthy volunteers

Whole blood samples were purchased from the Etablissement Français du Sang (EFS) (n = 50 different donors, aged 21 to 65, 39 men and 11 women). According to EFS standardized procedures for blood donation, written informed consent was obtained from HVs (healthy volunteers) and personal data for blood donors were anonymized at time of blood donation and before blood transfer to our research lab. This work belongs to a global study on ICU-induced immune dysfunctions which has been approved by our Institutional Review Board for ethics (“Comité de Protection des Personnes Sud- Est II”, Ref 2014–003).

### Cell isolation and seeding

PBMC (peripheral blood mononuclear cells) were obtained by Ficoll gradient centrifugation. Monocyte depleted PBMC were obtained using RosetteSep™ Human Monocyte Depletion Cocktail (Stem Cell, Vancouver, Canada), according to manufacturer’s instructions. T cells were isolated through negative selection using the RosetteSep™ Human T Cell Enrichment Cocktail (Stem Cell), according to manufacturer’s instructions. Monocytes were isolated through positive CD14 selection, using CD14 MicroBeads, MS columns and MiniMACS™ Separator, according to manufacturer’s instructions (Miltenyi Biotec, Bergisch Gladbach, Germany).

### Cell culture

Cells were diluted to working concentration (1x10^6^ cells/mL unless otherwise stated) in complete culture medium: RPMI 1640 (Eurobio, Les Ulis, France) supplemented with 10% SAB (AB human serum, Life technologies, Carlsbad, CA, USA), 2 mM L-Glutamine (Eurobio), 2 μg/mL Fungizone (Gibco, Life technologies), 20 U/mL penicillin and 20 μg/mL streptomycin (Peni 10000 UI/Strepto 10000 μg, Eurobio), and incubated at 37°C in humidified 5% CO_2_ atmosphere.

#### LPS priming

Cells were treated overnight with either medium or various concentration of LPS (from 0.01 to 1000 ng/mL) mix of gel filtration chromatography purified LPS from *Escherichia coli* O111:B4, O55:B5, O124:B8, Sigma Aldrich, Saint Louis, MO, USA).

#### IFNγ treatment

Following LPS priming, PBMC were washed with PBS, re-suspended in complete medium, treated with either medium or 100 ng/mL of human recombinant interferon gamma-1b (IFNγ, Immukin, Boehringer Ingelheim, Germany) and incubated at 37°C in humidified 5% CO_2_ atmosphere. For proliferation assay, T cell stimulant was added simultaneously with IFNγ.

### T cell proliferation assay

T cells were stimulated 72h at 37°C in humidified 5% CO_2_ atmosphere with one of the following stimulants: anti-CD2/CD3/CD8 antibodies-coated beads (αCD2/3/28-Abs coated beads, T cell activation/expansion kit, Miltenyi Biotec, 1 bead for 2 cells), 4 μg/mL phytohemagglutinin (PHA, Oxoid), 25 ng/mL anti-CD3 antibody (OKT3, mouse monoclonal IgG2a,κ, Tonbo Biosciences, San Diego, CA, USA).

T cell proliferation was then evaluated using the Click-iT® flow cytometry assay (LifeTechnologies, Carlsabad, CA, USA), as previously described [8]. Flow cytometry analyses were performed on a Navios flow cytometer (Beckman Coulter). CD3^+^ cells were first selected among total events based on a monoparametric CD3-allophycocyanin (APC) histogram (APC labeled anti-CD3 antibody, mouse monoclonal IgG1, clone UCHT1, Beckman Coulter). Then the percentage of EdU^+^ cells among CD3^+^ cells were measured on a monoparametric EdU-AF488 histogram. For every experiment, a minimum of 2.5x10^3^ CD3^+^ cells was recorded. Data were analyzed using Kaluza software (version 1.2, Beckman Coulter).

### IFNγ concentration dosage in cell supernatants

After PBMC culture, culture plates were centrifuged, supernatants harvested and stored at -80°C. All tested supernatants were thawed simultaneously and the Bio-Plex Pro™ Human Cytokine 8-plex Assay (Bio-Rad, Hercules, CA, USA) was performed on 50μL of each supernatant in duplicates, according to manufacturer’s instructions, and processed with a BioPlex 200 (Bio-Rad). Results were analyzed with the BioPlex Manager software 6.1.

### Flow cytometry immunophenotyping

Multiparametric flow cytometry panels were used to characterize expressions on monocytes and lymphocytes of various receptors. Antibodies were: PC7 (PE (phycoerythrin)-cyanin7) labeled anti-CD64 antibody (mouse monoclonal IgG1, clone 22,Beckman Coulter), PB (pacific blue) labeled anti-CD14 antibody (mouse monoclonal IgG22a, clone RMO52, Beckman Coulter), PE-labeled anti-CD86 antibody (mouse monoclonal IgG2b,κ, clone IT2.2 Biolegend, San Diego, CA, USA), Allophycocyanin-labeled anti-CD80 antibody (mouse monoclonal IgG1, clone MAB104, Beckman Coulter), PC7 labeled anti-HLA-DR antibody (human leukocyte antigen-DR) (mouse monoclonal IgG2aκ, clone L243, Biolegend), PE labeled anti-PD-1 antibody (goat polyclonal IgG, R&D systems, Mineapolis, MN, USA), PC7 labeled anti-CD28 antibody (mouse monoclonal IgG1, clone CD28.2, Beckman Coulter), APC-labeled anti-CTLA4 antibody (mouse monoclonal IgG1κ, clone L3D10, Biolegend, San Diego, CA, USA), and PB labeled anti-CD3 antibody (mouse monoclonal IgG1, clone UCHT1, Beckman Coulter). Corresponding isotypic antibodies labeled with the same fluorophores and purchased from the same suppliers were used as controls. Regarding monocyte receptor evaluation, CD14^+^ cells were first selected among total events based on a bi-parametric CD14-PB/side scatter plot. Then the MFI (means of fluorescence intensity) of each parameter on CD14^+^ cells were measured on mono-parametric histograms. For every experiment, a minimum of 2.5x10^3^ CD14^+^ cells was recorded.

Regarding T cell receptor expression evaluation, CD3^+^ cells were first selected among total events based on a bi-parametric CD3-PB/side scatter plot. Then the MFI of each parameter on CD3^+^ cells were measured on mono-parametric histograms. For every experiment, a minimum of 2.5x10^3^ CD3^+^ cells was recorded.

### Statistics

For each individual, technical replicates (a minimum of 2 per measurement) were summarized by their means. Such individual values are presented in the figures. In addition, for each condition, individual values are summarized on the plots by a horizontal line representing the mean of a given condition. Non parametric Wilcoxon matched paired signed rank test was used to evaluate the significance of quantitative measurements (the effect of the priming of T cells, monocytes or both on T cell proliferative response to OKT3, the restorative effect of IFNγ on decreased lymphocyte proliferation after LPS priming). Differences between groups were considered statistically significant for *P*-values lower than 0.05. Regarding dose-response effect of LPS priming on T cell proliferative response or production of IFNγ, the goodness of fit of the dose-response curve obtained was assessed through fitting the experimental data to a dose-response model for an inhibitor. The adequacy of the fit is measured by the r^2^. An r^2^> 0.5 was considered as a strong adequacy between observed data and the model therefore showing a dose-response effect. Anova analysis was performed to evaluate (i) the impact of LPS stimulation and the dose of LPS on T cell proliferative response; (ii) the impact of LPS stimulation, stimulation time, and interaction of both on monocyte expression of HLA-DR, CD64, CD86 and CD80. A statistically significant impact of the variables was considered for *P*-values lower than 0.05.

## Results

### Decreased lymphocyte effector functions after LPS priming

Sepsis-induced lymphocyte anergy is characterized by decreased effector functions (*i*.*e*. proliferation and cytokine production) in response to *ex vivo* stimulation [7,9]. Therefore, we tested the effect of LPS priming (mimicking initial Gram negative infectious challenge) on lymphocyte effector functions after incubation with different stimuli (PHA, anti-CD2/CD3/CD28-coated beads and OKT3) on cells from healthy donors [10–12].

**Fig 1:**
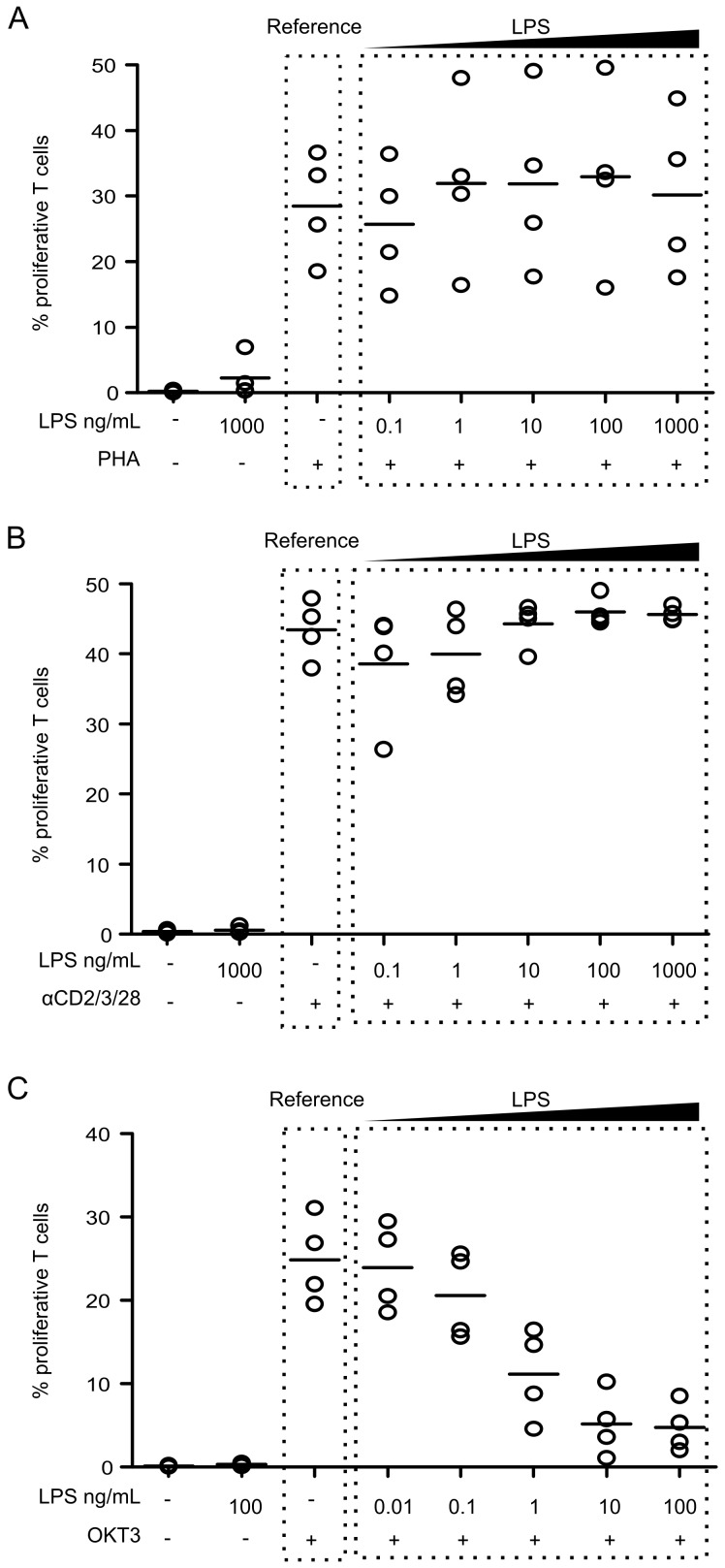
LPS dose-dependently decreases T cell proliferative response to OKT3. PBMC were primed overnight with LPS concentrations ranging from 0.01 ng/mL to 100 ng/mL (in case of OKT3 stimulation) or from 0.1 ng/mL to 1000 ng/mL (in case of anti-CD2/CD3/CD28-coated beads and PHA stimulations). Then PBMC were stimulated with 4 μg/mL PHA (**A**), 0.5x10^6^ anti-CD2/CD3/CD28-coated beads/mL (**B**), or 25 ng/mL OKT3 (**C**) for 72h. T cell proliferation was measured by flow cytometry and results are expressed as percentages of proliferating cells among total T cells. Each experiment was performed on n = 3 to 4 HV. Technical replicates were summarized by the mean for each individual. For each condition, individual values are summarized on the plots by a horizontal line representing the mean of a given condition.

First, lymphocyte proliferative response was evaluated. We did not observe any effect of LPS-priming on PHA- or anti-CD2/CD3/CD28-coated beads-induced lymphocyte proliferation independently of LPS concentration used during the priming phase (Fig 1A and 1B). In contrast, LPS priming induced a strong decrease in T cell proliferative response after OKT3 stimulation (Fig 1C). Importantly, this decrease was dose-dependent (*r* ^2^ = 0.78) of LPS concentration (Fig 1C).

**Fig 2:**
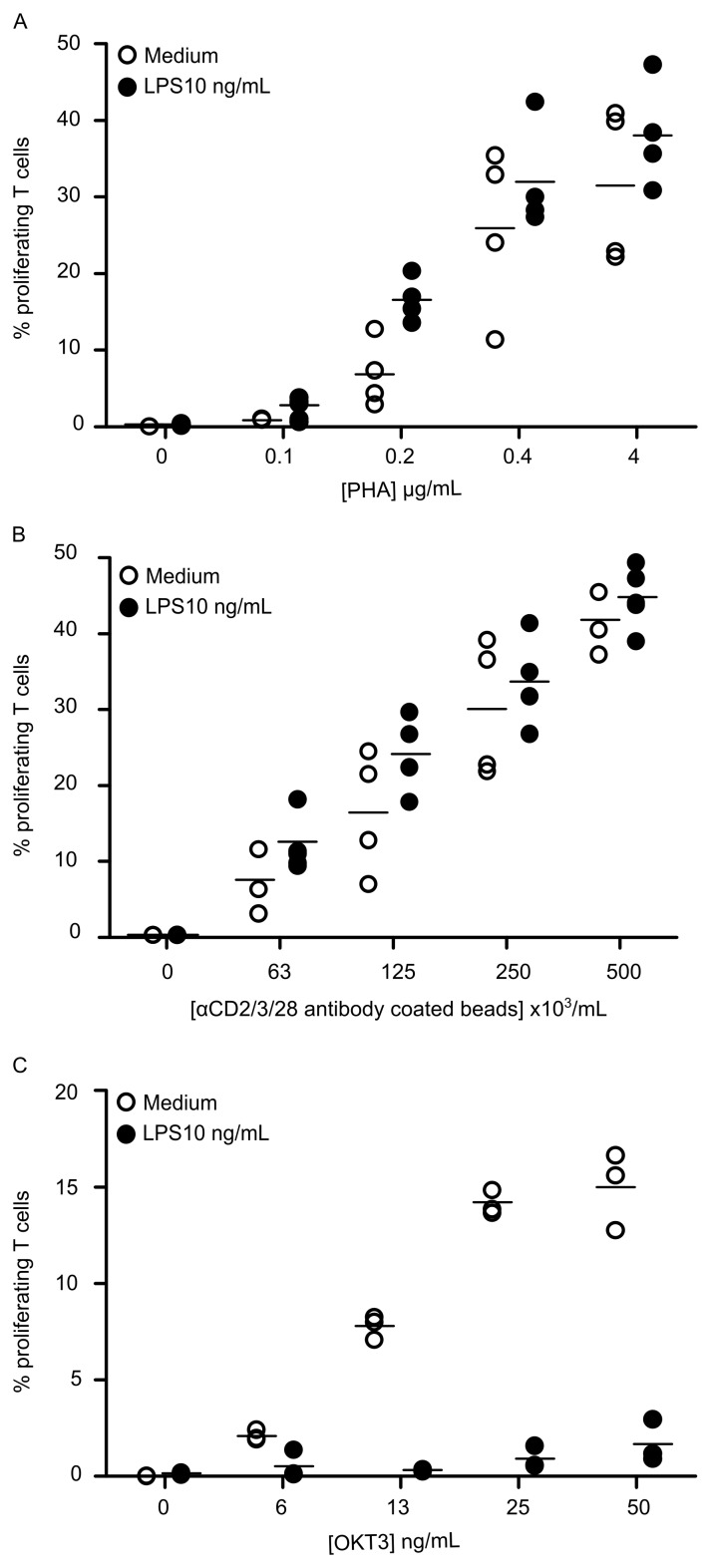
LPS decreases only T cell proliferative response to OKT3. PBMC were first treated with medium (white points) or primed with 10 ng/mL LPS (black points), then cells were stimulated with 0.1 to 4 μg/mL PHA (**A**), or 63x10^3^ to 500x10^3^ anti-CD2/CD3/CD28-coated beads /mL (**B**), or 5 to 50 ng/mL OKT3 (**C**) for 72h. T cell proliferation was measured by flow cytometry and results are expressed as percentages of proliferating cells among total T cells. Each experiment was performed on *n* = 4 HV. Technical replicates were summarized by the mean for each individual. For each condition, individual values are summarized on the plots by a horizontal line representing the mean of a given condition.

In order to exclude a putative dose effect of proliferation stimuli on these initial observations; we tested the effect of LPS priming on lymphocyte proliferation induced by lower stimuli concentrations. As shown in Fig 2A and 2B, no effect of LPS priming was observed on lymphocyte proliferation induced by PHA or anti-CD2/CD3/CD28-coated beads regardless of their concentrations levels. In marked contrast, after LPS priming, we observed a complete suppression of T cell proliferative response induced by OKT3 (*P*<10^−8^), for every tested concentration (Fig 2C).

**Fig 3:**
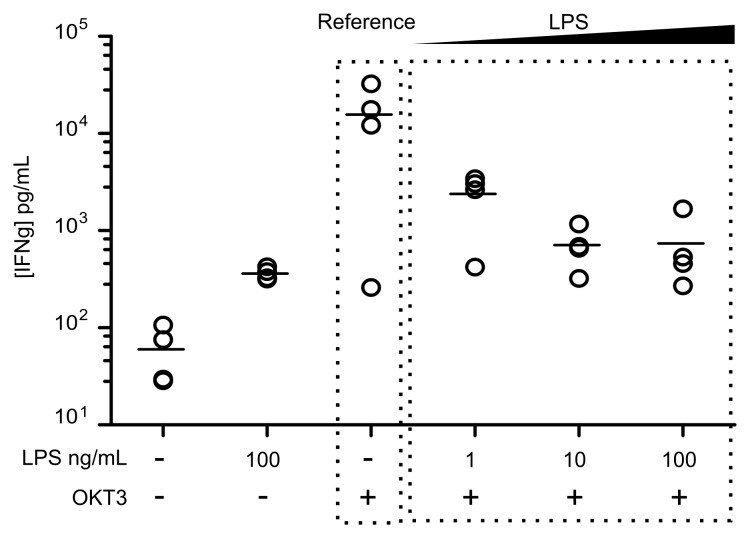
LPS dose-dependent reduction of IFNg secretion by T cells in response to OKT3 stimulation. PBMC were primed overnight with 1 ng/mL, 10 ng/mL or 100 ng/mL LPS. Then PBMC were stimulated with 25 ng/mL OKT3 for 72h before the surpernatant was recovered for IFNγ measurement. Each experiment was performed on *n* = 4 HV. Technical replicates were summarized by the mean for each individual. For each condition, individual values are summarized on the plots by a horizontal line representing the mean of a given condition.

Based on these preliminary results, we evaluated another important aspect of sepsis-induced lymphocyte anergy, the decreased cytokine production [13,14]. Hence, IFNγ concentrations were measured in culture supernatants of LPS-primed PBMCs after OKT3 stimulation. As shown in Fig 3, in addition to the reduced proliferation, we also observed a dose-dependent (*r* ^*2*^ = 0.53) effect of LPS priming on the reduction of IFNγ secretion induced after OKT3 stimulation.

Therefore, in this first set of experiments, we have shown that OKT3-induced lymphocyte proliferation and IFNγ production were altered after LPS priming *ex vivo*.

### Monocytes mediate the decreased lymphocyte proliferation after LPS priming

In order to understand the putative mechanisms leading to decrease T cell proliferation, we investigated which cell populations could be involved in LPS-priming effect. In particular, we wanted to know if LPS priming acts directly on T cell to affect their proliferation or may require interactions with other cells such as antigen presenting cells (APC). This aspect has never been investigated before.

**Fig 4:**
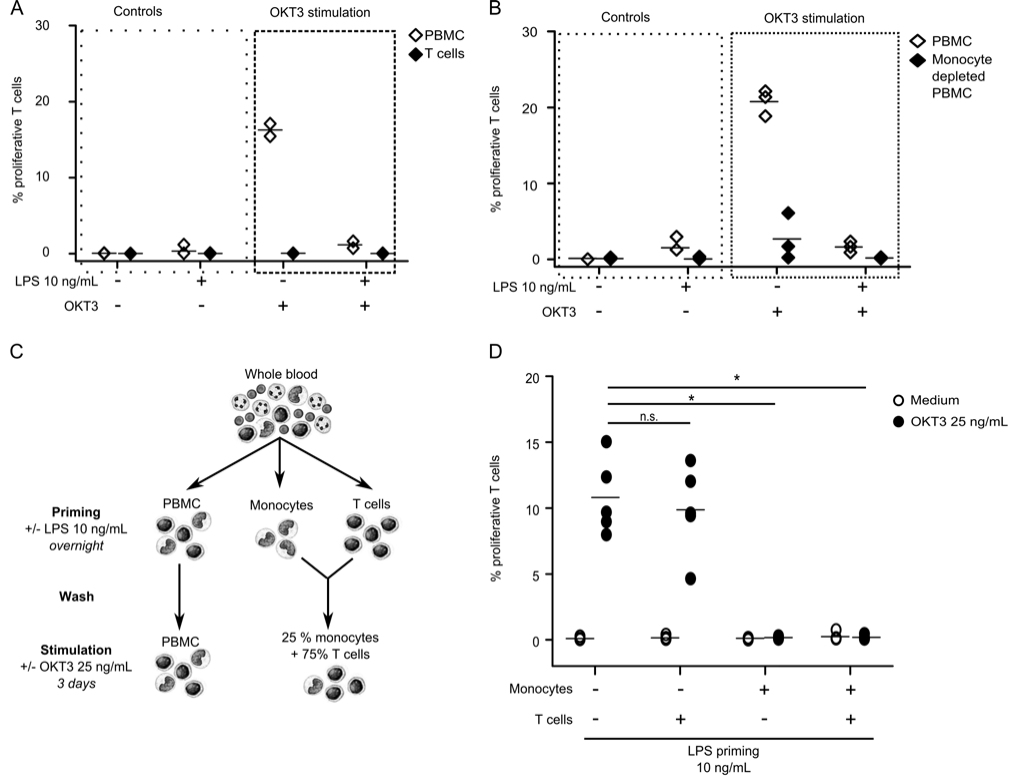
Monocytes mediate LPS induced decrease of T cell proliferative response to OKT3. PBMC (open diamonds) and T cells (**A**—black diamonds) or monocyte depleted PBMC (**B**- black diamonds) were, first, primed overnight by 10 ng/mL LPS, then, stimulated 72h with 25 ng/mL OKT3. T cell proliferation was measured by flow cytometry and results are expressed as percentages of proliferating cells among total T cells. This experiments were performed on *n* = 2 or 4 HV. Technical replicates were summarized by the mean for each individual. For each condition, individual values are summarized on the plots by a horizontal line representing the mean of a given condition. **C-** T cells and monocytes were isolated from each HV and were incubated separately overnight in medium alone or with 10 ng/mL LPS. After thorough PBS wash of each fraction, primed or unprimed T cell fractions were mixed with primed or unprimed monocyte fractions (0.5x10^6^ monocytes/mL and 1.5x10^6^ T cells–every combination was tested), and stimulated or not with 25 ng/mL OKT3 for 72h before T cell proliferation measurement. T cell proliferation was measured by flow cytometry and results are expressed as percentages of proliferating cells among total T cells. Similar experiment with total PBMC from each HV was performed in parallel as control. **D-** Results on purified monocytes and lymphocytes are presented. The different conditions of LPS priming of monocytes and / or T cells are underlined under the graph. Open circles represent results for cell culture conditions with no T cell stimulation. Black circles represent results after 25 ng/mL OKT3 stimulation for 72h. The experiment was performed on *n* = 5 HV. Technical replicates were summarized by the mean for each individual. For each condition, individual values are summarized on the plots by a horizontal line representing the mean of a given condition.

Therefore, the effect of LPS priming on OKT3-induced proliferation was first tested on isolated T cells in comparison with PBMCs from the same donor. We did not observe any evidence that OKT3 induces proliferation on isolated T cells. In the contrary, PBMCs stimulated with OKT3 were induced to proliferate, but LPS priming decreased their proliferative responses (Fig 4A). This result shows that OKT3 stimulation requires the presence of accessory cells to induce T cell proliferation in accordance with the literature [15–17].

Among accessory cells, we next investigated the role of monocytes in our model. T cell proliferative responses induced by OKT3 were thus evaluated in monocyte-depleted PBMCs cultures. Consistently with our previous results, we observed that OKT3 stimulation did not induce any T cell proliferation if monocytes were depleted, while PBMC proliferations as well as the inhibitory effect of LPS priming on these proliferative responses from the same donors were conserved (Fig 4B). This highlights the central role of monocytes in this model and suggests that the reductive effect of LPS priming on OKT3-induced lymphocyte proliferation might be mostly mediated by monocytes.

To confirm this aspect, we designed experiments in which purified T cells and purified monocytes were first primed separately by LPS before being co-cultured to evaluate OKT3-induced proliferation (Fig 4C). We observed that T cell proliferative response to OKT3 was decreased only when monocytes where primed with LPS, while LPS priming of purified T cells had no effect (Fig 4D). This confirms that LPS priming affects monocytes to reduce OKT3-induced T cells proliferation in our model.

**Fig 5:**
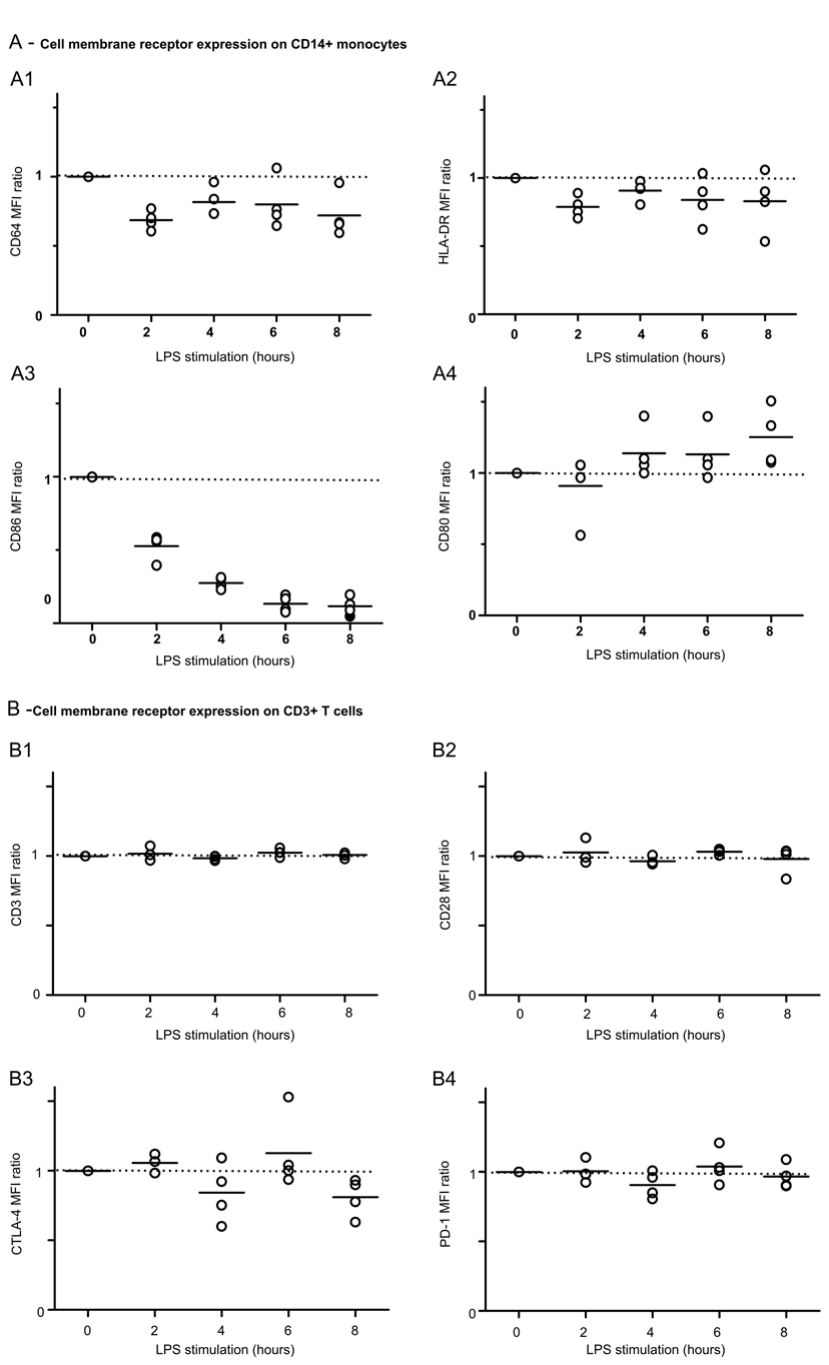
LPS priming alters monocyte T cell accessory cell function but not T cell receptor expression. PBMC were treated with medium or 10 ng/mL LPS and flow cytometry phenotyping was performed every 2h for 8h. Four markers were studied on monocytes (**A**): CD64 (**A1**), HLA-DR (**A2**), CD86 (**A3**) and CD80 (**A4**). For each marker, mean fluorescence intensities (MFI) on CD14^+^ cells were measured. Four markers were studied on T cells (**B**): CD3 (**B1**), CD28 (**B2**), CTLA-4 (**B3**) and PD-1 (**B4**). For each marker, mean fluorescence intensities (MFI) on CD3^+^ cells were measured. MFI ratio were calculated as follow = MFI of LPS treated cells divided by the MFI of the medium-treated cells at the same time point. The experiments were performed on *n* = 4 HV. Technical replicates were summarized by the mean for each individual. For each condition, individual values are summarized on the plots by a horizontal line representing the mean of a given condition.

Finally, to evaluate how LPS priming of monocytes could participate in their reduced capacity to induce OKT3-mediated proliferation; we measured CD64, HLA-DR, CD86, and CD80 expressions on these cells during incubation with LPS. Indeed, it has been shown that co-stimulations through MHC II molecules, CD80 and CD86 co-receptors as well as FcγRI (CD64) presentation participate in OKT3-induced proliferation[15,17,18]. We observed that CD64 and CD86 expressions were significantly decreased on monocytes during LPS priming (P<0.05) whereas CD80 expression was not modified (Fig 5A). HLA-DR was not decreased during priming (Fig 5A2) but significantly decreased during the following days (data not shown). This suggests that these decreased co-stimulatory molecules expressions after LPS priming may participate in the lowered T cell proliferative response observed after OKT3 stimulation of primed PBMCs. To note, LPS priming had no effect on CD28, CTLA4, PD-1 and CD3 expressions on T lymphocytes (Fig 5B).

Taken together, these results showed that LPS priming acts on monocytes to impair OKT3-induced lymphocyte proliferation through an alteration of monocytes’ accessory cell functions. Importantly, in this model, LPS priming does not appear to have any direct effect on lymphocytes.

### *Ex vivo* restoration of lymphocyte proliferation after LPS priming

Finally, we tested the effect of an immunoadjuvant therapy (*i*.*e*. IFNγ), known to restore monocyte functions, on lymphocyte proliferative response and monocytes accessory cell functions *ex vivo* after LPS priming.

**Fig 6:**
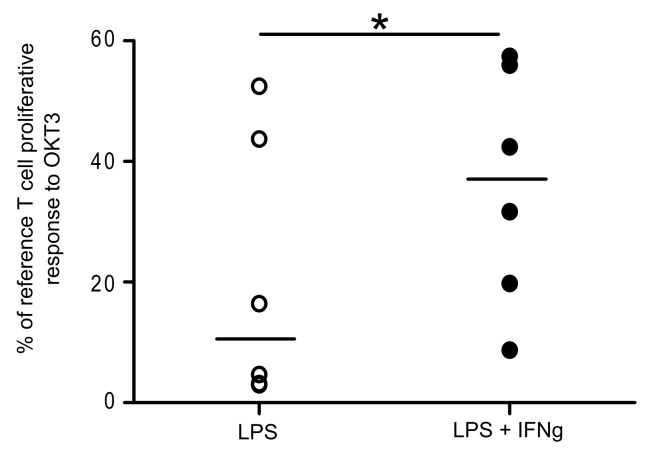
IFNγ partially restores T cell proliferative response to OKT3. PBMC were treated or not overnight with 10 ng/mL LPS, washed and then treated simultaneously or not with 100 ng/mL IFNγ and 25 ng/mL OKT3 for 72 h before T cell proliferation measurement (Black circles: cells primed with LPS and treated with IFNγ+OKT3; open circles: cells primed with LPS and treated with OKT3 but with no IFNγ treatment). T cell proliferation was measured by flow cytometry and results are expressed as percentages of proliferating cells among total T cells. In addition, results are normalized versus proliferation measured after OKT3 stimulation without any LPS priming or IFNγ treatment (control condition). Results are expressed as percentages to the reference proliferative response. The experiments were performed on *n* = 6 HV. Technical replicates were summarized by the mean for each individual. For each condition, individual values are summarized on the plots by a horizontal line representing the median of a given condition.

**Fig 7:**
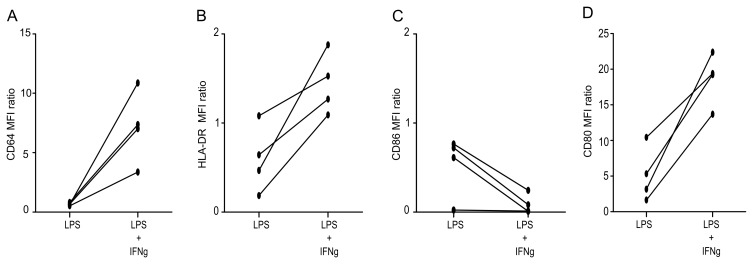
IFNγ partially restores monocyte accessory cell function. PBMC were incubated for 12h with medium or 10 ng/mL LPS, washed, and treated with medium or 100 ng/mL IFNγ. MFI of either CD64 (**A**), HLA-DR (**B**), CD86 (**C**) or CD80 (**D**) on CD14^+^ cells were measured, and MFI ratios calculated by dividing the MFI of treated cells by the MFI of control cells (no treatment). The experiment was performed on *n* = 4 HV. Technical replicates were summarized by the mean for each individual.

As shown in Fig 6, we observed that IFNγ treatment significantly improved OKT3-induced T cell proliferative response after LPS priming (while IFNγ treatment alone did not affect T cell proliferative response to OKT3, data not shown). In addition, we observed that this was associated with increased CD64, HLA-DR and CD80 monocyte expressions (Fig 7).

This confirms that, after LPS priming, IFNγ treatment can improve T cell proliferative response through the restoration of monocyte’s accessory functions.

## Discussion

The pathophysiology of sepsis associates a complex interplay between pro- and anti-inflammatory responses alternatively predominating overtime [7]. Indeed, while initial overwhelming pro-inflammatory response occurs after septic shock (responsible for organ dysfunctions and early deaths); anti-inflammatory mechanisms rapidly develop in patients that survive this initial peak of cytokine production. If persisting overtime, such compensatory immune response might become deleterious, as it could lead to major immune alterations [13,14]. Indeed, during sepsis, immune dysfunctions affecting both the innate and the adaptive immune responses have been described. Importantly, several clinical studies showed that the intensity and duration of sepsis-induced immune alterations are associated with increased risk of death and HAI. This represents the rational for novel clinical trials testing immuno-adjuvant therapies in septic patients that present with such immune alterations [7].

In particular, sepsis-induced lymphocyte alterations are characterized by (i) a marked decrease in circulating cell number due to increased apoptosis, (ii) phenotypic alterations such as increased co-inhibitory receptor, decreased CD3 and co-stimulatory receptor expressions, (iii) reduced TCR diversity, (iv) increased proportion of regulatory T cells and, (iv) functional alterations such as decreased proliferation and cytokine production *ex vivo* [4,5,14,19]. This last aspect is characteristic of lymphocyte anergy / exhaustion with similarities with lymphocyte alterations described after chronic viral infections [3]. Importantly, several observational studies in clinic showed an association between the intensity and duration of sepsis-induced lymphocyte alterations and increased risk of death or HAI in patients [13,20,21].

Mechanisms leading to such lymphocyte alterations are not completely understood. However, preliminary studies in patients showed that both antigen-specific and mitogen-mediated lymphocyte responses are altered after septic shock. Indeed, Manjuck *et al*. showed the decreased proliferative response to recall antigen stimulations (tetanus toxoid or candidin) in patients with sepsis in association with a loss of HLA-DR and CD86 expressions on monocytes [22]. In addition, the decreased proliferation of septic patients’ lymphocytes after a non-specific direct stimulation through TCR (anti-CD2/CD3/CD28-Abs coated beads [23]) or to mitogen stimulation (PHA [24,25]) has been described. Therefore, both intrinsic lymphocyte alterations as well as antigen-presenting cells dysfunctions are most likely involved in sepsis-induced lymphocyte dysfunctions. In that context, the exploration of these aspects *ex vivo* could help to understand the pathophysiology of sepsis-induced lymphocyte dysfunctions. This represents the goal of the current study.

In this set of experiments, we investigated whether LPS priming (mimicking initial infectious challenge) could directly act on lymphocytes to affect their effector functions. Indeed, although TLR activation is the hallmark of the innate immune response, recent evidence demonstrates that adaptive immune cells use these innate signaling pathways as well [26].

We showed that LPS priming of PBMCs led to a reduced T cell proliferative response and altered IFNγ secretion only after OKT3 stimulation, while no effect of LPS priming was observed on PHA and anti-CD2/CD3/CD28 coated beads-induced lymphocyte responses. This suggests that LPS does not act directly on T cells to reduce their effector functions but may impact lymphocyte responses through their interactions with APCs even in a non-antigen specific manner. Here we present evidence for the indirect effect of LPS on T cells. We provide the first report showing that only LPS priming of monocytes led to decreased T cell proliferative response (although the possible impact of positive versus negative purification techniques of monocytes should be evaluated) as opposed to direct LPS priming of purified lymphocytes. Importantly, LPS priming effect was associated with decreased HLA-DR, CD86 and CD64 expressions on monocytes but no change in CD28, CTLA4, PD-1 or CD3 expressions on T cells. Finally, IFNγ stimulation of monocytes restored their accessory functions (*i*.*e*. increased CD64, CD80 and HLA-DR expressions) and T cell proliferative response to OKT3.

Importantly, this highlights that, depending on the stimuli used to induce lymphocyte effector functions, APC-mediated T cell dysfunctions or intrinsic lymphocyte alterations could be evaluated. Indeed, the 3 stimuli evaluated in the current study use different mechanisms to promote T cell effector functions. PHA is a lectin that aggregates TCRs at T cell surface, thus leading to lymphocyte activation [27,28]. Anti-CD2/CD3/CD28-coated beads mimic TCR stimulation and induce T cell activation with no need for help from APC. Finally, OKT3 is an anti-CD3 antibody that stimulates TCR with the need for an interaction with APC but in a non-antigen specific manner. Indeed the mitogenic activity of OKT3 clone is dependent on monocyte accessory functions. In particular, it correlates with the capacity of the Fc portion of this antibody to interact with FcRs that are present on phagocytes [16,29] and requires interactions with co-stimulatory molecules such as CD80/CD86 or even HLA-DR [17,18]. This supports the importance to evaluate in parallel several stimuli in clinic to decipher intrinsic versus extrinsic/APC-mediated lymphocyte dysfunctions.

Our results show that LPS priming does not directly impact lymphocyte effector functions in our model. Numerous studies have investigated the effect of TLR4 ligands such as LPS on lymphocyte effector functions [26]. Several different models were evaluated (*in vivo* experiments in mice [30–33], *ex vivo* studies on purified cells [34,35], on various cell populations (cell lines [36]; PBMC [37,38], purified CD4^+^ T cells [39–41]) with various experimental conditions (for example: LPS dosage ranging from 10ng [30] to 250μg [33]). In addition, a number of different read-outs were evaluated: (i) cell proliferation [42]; (ii) cell activation [30], (iii) cell survival / apoptosis [33], (iv) cytokine production [41] or (v) cell adherence [43]. Depending on the experimental set-up, either an activating or an inhibitory effect or the absence of any effect of LPS on lymphocytes was observed. In particular, most of the studies that observed a stimulatory effect of LPS on T cells were performed in mice *in vivo*; a different experimental set-up compared with our current study. Indeed, if considering only studies performed with primary human cells *ex vivo*, results are less clear. Indeed, while some authors did not observe any effect of LPS on T cells [40]; some authors reported either a direct stimulating effect of LPS on T cells [38], a stimulating effect mediated by monocytes [37] or, conversely, a direct [44] or monocyte-mediated [45] inhibitory effect. Here again, different experimental conditions (work on purified cells or mixed cell populations, different LPS dosages …) could explain these discrepant results. In addition and most importantly, studies showing an immunoadjuvant effect of LPS on T cell functions evaluated the simultaneous incubation of LPS with T cell stimuli. In our current experimental protocol, we tested whether a priming phase of T cells with LPS could impact a subsequent stimulation of lymphocytes. This experimental design was derived from the model of endotoxin tolerance that has been shown to recapitulate ex vivo monocyte-induced immune dysfunctions [10].

With that said, our results are in accordance with previous studies that reported no direct effecy of LPS on T cells [39–41,46]. In particular, Komai-Koma *et al*. did not observe any effect of LPS on proliferation and cytokine production of anti-CD3 activated human T cells [40]. To note, our current experimental protocol (EdU incorporation after 72h of stimulation) does not permit the evaluation of an “early” stimulating effect of LPS on TCR-induced proliferation. This specific point now needs to be evaluated in a dedicated study with the use of another read-out for cell proliferation such as CFSE incorporation.

Interestingly, stimulation of other TLRs such as TLR2 has been repeatedly reported to improve T cell proliferation and cytokine production in mice and humans [26,40]. Therefore, despite our negative results, such direct effect of TLR ligands in sepsis-induced lymphocyte alterations could not be excluded and this remains to be evaluated by, for example, studying the effect of other TLRs and PAMPs or DAMPS stimulations. For example, Domingez-Villar *et al*. set-up a model of T cell exhaustion by treating CD4^+^ T cells with Imiquimod, a TLR7 agonist, and observed a decrease of proliferation and of IFNγ production in response to soluble anti-CD3 and anti-CD28 stimulations. This model was developed to study aspects of lymphocyte exhaustion after chronic viral infections (*i*.*e*. HIV) which shares characteristics with sepsis induced T cell anergy [47].

In our model, LPS acts on monocytes/APCs to reduce their capacity to activate T cells. This is clearly relevant with the pathophysiology of sepsis in which monocyte alterations have been shown to participate in sepsis-induced lymphocyte dysfunctions [22,48]. Other groups have reported similar results. For example, the study by Yaqub *et al*. showed, in a whole blood model, that LPS stimulation could reduce T cell effector functions induced by TCR/CD3 or SEB stimulations through the production of PGE2 [49,50]. Interestingly in a second study by this group, the authors showed that this effect was indeed mediated by monocyte production of PGE2 after LPS stimulation [50]. Similarly, Wolk *et al*. showed that LPS priming of mononuclear cells down-regulated MHC II molecules and CD86 expressions, as observed in the current study, in association with diminished T-cell proliferation and IFNγ production in response to presentation of different recall antigens [45]. In the current study, we extend these previous results by showing that not only antigen-specific but also antigen non-specific monocyte-mediated lymphocyte activation (after OKT3 stimulation) is altered after bacterial challenge; an aspect that was not evaluated in Wolk’s work. This is important since it allows for the study of monocyte accessory cell functions / co-stimulatory molecule expressions role in sepsis-induced lymphocyte alterations. This might be of major interest in a context of already starting clinical trials testing treatments targeting such immune checkpoints in clinic [51,52], and knowing that such therapies are considered in the treatment of septic shock patients that present with immune alterations [53,54]. In addition, from a technical perspective, the proliferative response induced by OKT3 is of a larger proportion than that induced by recall antigens, which facilitates its evaluation in particular in a case of a reduced effect.

Finally, similar results have also been observed after monocyte stimulation with other TLR ligands. For example, Nakagawa *et al*. stimulated PBMC of healthy volunteers with peptidoglycan prepared from *S*. *aureus* and observed a loss of T cell IFNγ production and proliferation in response to fixed anti-CD3 antibody plus soluble anti-CD28 antibody stimulation [55]. They demonstrated that this effect required cell-to-cell contact of T cells with peptidoglycan-stimulated monocytes.

Notably, our results also suggest that therapies that restore APC functions (such as IFNγ as tested here) probably have a positive effect not only on innate immunity but also on adaptive immune cells after sepsis. This may be of major importance and may have participated in the positive effect observed for such treatments in the preliminary clinical studies testing these interventions [56–58]. However, this aspect has not been investigated in these clinical trials, but should be considered in the forthcoming clinical studies testing immunoadjuvant therapies.

However, it is highly possible that other mechanisms directly targeting lymphocytes could also participate in sepsis-induced lymphocyte alterations. One can speculate on the roles of immunosuppressive cytokines, apoptosis increasing molecules and/or glucocorticoids. However, this still deserves to be demonstrated in clinic, in particular regarding the respective parts of direct versus indirect mechanisms of sepsis-induced lymphocyte alterations. To that purpose and as discussed above, we would recommend that both direct T cell stimuli (such as PHA) and molecules that require interactions with monocytes to induce lymphocyte proliferation (such as OKT3) should be evaluated in parallel in the forthcoming clinical studies evaluating sepsis-induced lymphocyte alterations in clinic.

In this study, we show that LPS priming does not directly impact lymphocyte effector functions. However such stimulation could act on monocytes/APCs to reduce their capacity to activate T cells. Interestingly, this negative regulation of lymphocyte proliferation was restored *ex vivo* by APC-targeting drugs such as IFNγ. We think that our results recapitulate *ex vivo* the part played by sepsis-induced innate immune cell dysfunctions in sepsis-induced lymphocyte alterations. Further work is now necessary to study the respective parts of direct versus indirect mechanisms of sepsis-induced lymphocyte alterations in clinic and to develop complementary *ex vivo* models recapitulating other mechanisms directly leading to sepsis-induced lymphocyte alterations. The ultimate goal would be to be able to assess *ex vivo* the various candidate molecules targeting these dysfunctions.
